# 

**Published:** 1921-07-30

**Authors:** 


### A Tuberculosis Scheme Criticism.

The new tuberculosis scheme proposed by the Ministry of Health does not meet with the support of all local authorities. The Bethnal Green Borough Council, for instance, reports that the Advisory Committee of the City of London Hospital for Diseases of the Chest accepts the view of the Tuberculosis Officer that a large proportion of the patients can be more satisfactorily treated by the specialist staff at the dispensary, and that the effect of diminishing such treatment will be to deter patients from coming to the dispensary, and will thus actually diminish the educational value of the dispensary's work. The proposed restrictions of the Ministry—that dispensary treatment should, as a rule, be limited to patients whose continued treatment requires special knowledge or technical skill and to those who are unable to obtain other adequate medical attendance—are deemed by the Hospital Advisory Committee to be a retrograde step, and would not be to the ultimate benefit of persons suffering from tuberculosis. The medical officer of health agrees, saying that the ministerial proposals would diminish the efficiency of the tuberculosis scheme and would not effect any real economy.

### The Book Market.

D. Appleton & Co.: "The Principles and Practice of Surgery." By Hermun A. Haubold, M.D. (New York University.) Cloth. Illustrated. 2 vols. 84s. net.

J. B. Lippincott Co.: "International Clinics." Vol. IV. Thirtieth Series. "Gynæcology." By B. M. Anspach, M.D. 42s. net.

### The College of Nursing.

**(Limited by Guarantee.)**

### LONDON CENTRE.

(Secretary, Miss BOMPAS, 7 Henrietta Street, W. 1.)

MISS BILLINGSHURST, a London Centre member who has gained the Sister-Tutor Scholarship at King's College for Women, given by the London Centre members, has written to the Secretary to express her very grateful thanks to the members of the London Centre. She assures them that she will do her very utmost to make the best use of this privilege. London Centre members on their part will undoubtedly watch Miss Billinghurst's career at King's College with interest.

### CARDIFF CENTRE.

(Hon. Secretary, Miss HITCH, Anthony House, 30 Newport Road.)

ON Monday, July 25, the members of the Centre were entertained by Sir William and Lady Thomas at a garden party at Birchwood Grange, Penylan. Among the guests were some of the influential residents of Cardiff, as one of the objects of the gathering was to spread knowledge and arouse interest in the College of Nursing and its aims, and especially in the Cardiff branch of it. Miss Todd, when proposing a vote of thanks to Sir William and Lady Thomas, briefly stated the necessity for a nurses' club in Cardiff, which want it is the aim and hope of the Centre to fill at no distant date. In spite of somewhat unsatisfactory weather conditions, a most enjoyable afternoon was spent, and a considerable number of nurses were present.

### GLASGOW CENTRE.

(Hon. Secretary, Miss MOSELEY, Western District Hospital, Oakbank.)

ON July 15, at the headquarters of the Centre, Miss Sheriff-Macgregor, Organising Secretary, gave an address on "The Work of the College and the Uses of Local Centres." She pointed out that the College is a democratic institution. The members themselves nominated whom they pleased to represent them on the Council, and voted for their representatives. The local Centres met the need for co-operation and centralisation, and could aid the members by forwarding the aims of the College for the nursing profession, and by helping individual members of the Centre in whatever way they felt to be most useful.

### Resignation of a Hospital President.

ON Monday afternoon, July 4, Mrs. Bridgeman, who has resigned the Chairmanship of the Florence Nightingale Hospital Committee, was presented with Rudyard Kipling's poems, a selection of R. L. Stevenson's works, and an illuminated address. The presentation was made for the staff by the matron, Miss Houghton, in the presence of the whole staff and some of the members of the Committee. Mrs. Bridgeman has served as Lady President for fifteen years, and her help and unfailing kindness will be greatly missed. A few appropriate words were said by the matron on presenting the books, and Mrs. Bridgeman responded in a short inspiring speech, in which she said how grieved she is at having to give up a work which she loves. After the presentation the visitors were invited to have tea on the roof, which looked its gayest with the flowers cultivated in the boxes. It was through Mrs. Bridgeman's efforts that the Florence Nightingale Hospital was rebuilt and opened free of debt in 1910. During the fifteen years she has been President she has worked unceasingly for the hospital and the staff and the welfare of the patients. Mrs. G. Burroughes has been elected to succeed her.

### Awards at University College.

THE following awards in the faculty of medical sciences have been made at University College: *Cluff Memorial Prize*: Katharine A. C. Gillie. *Anatomy*: Senior Class Gold Medal, L. Reuvid; Junior Class Silver Medal, Mary E. Tyars. *Physiology*: Senior Class Gold Medal, Katharine A. C. Gillie; Junior Class Silver Medal, Iris M. Harmer. *Organic and Applied Chemistry*: General Course Silver Medal, Marian G. Palmer.

### Passing Events and Topics.

### Child Welfare Fete at Chelsea.

THE Red Cross Infant Welfare Centre Council and the various societies which it co-ordinates will shortly be moving from 20 Berkeley Street to a new home at 117 Piccadilly, which has been presented by the Carnegie Trustees. In order to carry on its work at the highest pitch of usefulness and to make it cover as wide a field as possible funds are required, and with this purpose a Fête and Revel were held on Saturday, July 16, at the Royal Hospital Gardens, Chelsea. A happy feature of the opening ceremony was the singing by the Chelsea pensioners and a children's choir of "Land of hope and glory." A large number of well-known people were present, and patronised the various side-shows and entertainments. One of the most interesting was the display in Arab costume of tent-pegging by the mounted branch of the Metropolitan Police. Miss Gladys Cooper opened the Fête.

### A "National Whist-Drive."

FOR the benefit of St. Dunstan's and the National Institute for the Blind, a "National" whist-drive is to be held in the autumn. It is being organised on a comprehensive scale. Local, district, and county championships will be held, and the winners of these will take part in the final contest in London. Winners of local and district championships will receive prizes, and of county championships silver cups, while the winner of the final will receive a silver challenge cup and £1,000 in cash. The organiser of this national whist championship is at 306 Regent Street, W. 1, where all who are willing to help should communicate with him.

### Civic Education League

HEALTH and other social workers should be interested in the Summer School of Civics, which is being held at Guildford during the fortnight from July 30 to August 13. The courses of study are arranged to offer much interest to them, and, in addition to the courses of lectures, the programme provides exhibitions of modern and classical art, regional survey exhibitions, a loan exhibition of Guildford, old and new, and an exhibition of books on civics. There are also dramatic and musical entertainments and a series of public evening lectures. There are still some vacancies for students, who should write immediately to the Secretary, Miss Margaret E. Tatton, Leplay House, 35 Belgrave Road, S.W. 1.

### An Interesting Endowment.

IN appreciation of the action of the firm over their wages, the employees of Messrs. W. D. & H. O. Wills who served in the war subscribed a sum of £520, which has been used to endow a cot in No. 1 ward of the Bristol Royal Hospital for Sick Children. The tablet over the cot, which was lately unveiled, records not only the donors, but the motive of their gift, which deserves general recognition.

### Sixty-five Babies Saved.

THE number of infant deaths diminished at Newhaven in 1920 from 100 to thirty-five, and the Chairman of the Newhaven and District Nursing Association, at the annual meeting, justly attributed this fine result, largely to the efforts of the health visitor and mother-craft centre. Such facts cannot be too prominently brought before the public.

### Help in Appeal Work.

WE understand that the Roneo Co., Ltd., of High Holborn, are willing to give to hospitals, where good use can be made of it, one of their duplicators free of charge. Many London hospitals have been supplied in this way, and the machine is found to be most helpful in connection with appeals. This is the contribution of the Roneo Co. to the hospitals in place of sending donations.

### University College and Hospital Athletic Ground.

THE male students of University College are endeavouring to raise £2,000 before August 31 to complete the sum required for the athletic ground at Perivale for the purposes of the College and the hospital. Some fifteen and a quarter acres were acquired in 1907, and a further seven acres for extensions in 1920. With the necessary laying out of the land a sum of £6.000 was required, of which £4,000 has already been raised.

July 30, 1921] [Price Twopence

### The Hospital

**The College of Nursing (Limited by Guarantee)** 308

**Passing Events and Topics** ... ... ... ... 308

**RATES OF SUBSCRIPTION** (post free, payable in advance).

Three months, 3/3; six months, 6/6; twelve months, 13/-. (Should the cost of printing and paper as well as increased postage, necessitate alteration of these rates, the period paid for will be reduced proportionately on unexpired subscriptions.) THE HOSPITAL "Index" to Volume LXIX., October 1920 to March 1921, is now ready, price 1/1 per copy, post free. Address The Manager, THE HOSPITAL, "The Hospital" Building, 28/29 Southampton Street, Strand, London, W.C. 2.

THE FOREMOST AMERICAN HOSPITAL JOURNAL

Beautifully Printed—Abundantly Illustrated.

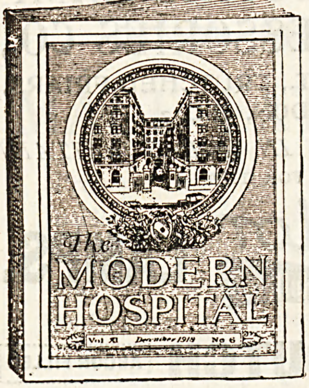

**THE MODERN HOSPITAL**

A 250-page monthly publication devoted to the architecture, equipment and administration of hospitals, sanatoriums, and allied Institutions.

**Authoritative and Widely Read.**

The Editorial Staff of THE MODERN HOSPITAL is composed of hospital executives of international reputation, whose experience covers every detail of the construction, equipment, organisation and administration of hospitals. This fact, combined with the large volume and practical character of hospital information furnished its readers, has given THE MODERN HOSPITAL unqualified recognition as the leading journalistic authority of the Western Hemisphere on all questions relating to institutional care of the sick, and has caused it to be regarded an indispensable aid by managing officials, members of medical, surgical and nursing staffs, dietitians and others representing practically all of the important hospitals and kindred institutions of the United States and Canada; its circulation extending also into more than twenty other countries.

**SPECIMEN COPIES MAILED FREE.**

A Specimen Copy of THE MODERN HOSPITAL will be mailed free of charge, fully post paid, to any address on request. The annual subscription price (foreign) is **$4.00.**

THE MODERN HOSPITAL PUBLISHING COMPANY, INC.,

22e ONTARIO STREET, CHICAGO, U.S.A.

